# Evaluation of a Subunit H5 Vaccine and an Inactivated H5N2 Avian Influenza Marker Vaccine in Ducks Challenged with Vietnamese H5N1 Highly Pathogenic Avian Influenza Virus

**DOI:** 10.1155/2010/489213

**Published:** 2010-06-27

**Authors:** Tze-Hoong Chua, Connie Y. H. Leung, H. E. Fang, Chun-Kin Chow, Siu-Kit Ma, Sin-Fun Sia, Iris H. Y. Ng, Stanley G. Fenwick, Cassandra M. James, Sin Bin Chua, Siang Thai Chew, Jimmy Kwang, J. S. M. Peiris, Trevor M. Ellis

**Affiliations:** ^1^School of Veterinary and Biomedical Sciences, Murdoch University, 90 South Street Murdoch, WA 6150, Australia; ^2^Agri-Food and Veterinary Authority of Singapore, 5 Maxwell Road, Singapore 069110; ^3^Department of Microbiology, The University of Hong Kong, Pathology Building, Queen Mary Hospital Compound, Pokfulam, Hong Kong; ^4^Temasek Life Sciences Laboratory, 1, Research Link, National University of Singapore, Singapore 117604

## Abstract

The protective efficacy of a subunit avian influenza virus H5 vaccine based on recombinant baculovirus expressed H5 haemagglutinin antigen and an inactivated H5N2 avian influenza vaccine combined with a marker antigen (tetanus toxoid) was compared with commercially available inactivated H5N2 avian influenza vaccine in young ducks. Antibody responses, morbidity, mortality, and virus shedding were evaluated after challenge with a Vietnamese clade 1 H5N1 HPAI virus [A/VN/1203/04 (H5N1)] that was known to cause a high mortality rate in ducks. All three vaccines, administered with water-in-oil adjuvant, provided significant protection and dramatically reduced the duration and titer of virus shedding in the vaccinated challenged ducks compared with unvaccinated controls. The H5 subunit vaccine was shown to provide equivalent protection to the other two vaccines despite the H5 antibody responses in subunit vaccinated ducks being significantly lower prior to challenge. Ducks vaccinated with the H5N2 marker vaccine consistently produced antitetanus toxoid antibody. The two novel vaccines have attributes that would enhance H5N1 avian influenza surveillance and control by vaccination in small scale and village poultry systems.

## 1. Introduction

Control of the H5N1 highly pathogenic avian influenza (HPAI) epizootic in village communities in Southeast and East Asia since 2003 has been difficult. Conventional control methods used for HPAI, including quarantine, enhanced biosecurity, and stamping-out are often not logistically possible in these villages where nutrition and livelihoods depend on low intensity poultry production. Use of vaccination against H5N1 avian influenza has become an important control tool in these settings [1]. All avian influenza control programs have the goals of protection from diseases, inhibition of further virus replication and transmission. Where vaccination is used, clinical disease may be prevented but ongoing surveillance is essential to monitor if the virus is still present or circulating in the avian population [1].

Ducks and other members of the Anatidae family are natural host species of influenza A viruses [2]. Prior to 2002, H5 or H7 subtype avian influenza viruses that were highly pathogenic for terrestrial poultry were generally non- or mildly pathogenic for ducks, but from late December 2002 circulating H5N1 HPAI viruses have been shown to be highly pathogenic for farmed and wild ducks and a range of other wild bird species [3–5]. H5N1 viruses isolated from the H5N1 HPAI epizootic that commenced in 2003 have shown some variation in virulence for ducks in experimental studies [4, 6]. In ducks challenged with 2003-2004 H5N1 viruses, some surviving ducks shed virus for a prolonged period of up to 17 days [7]. Apparently, healthy ducks may harbour H5N1 virus and shed virus for up to 17 days, thus becoming a potential source of infection for other poultry [7]. In many village communities in Southeast Asia, ducks are reared in large numbers as an adjunct to rice farming. In Thailand and Vietnam, spatial and statistical analyses demonstrated a significant association between H5N1 outbreaks the abundance of domestic ducks and rice-cropping intensity [8]. Infected ducks in some of these flocks were apparently healthy and did not show any disease signs, making clinical detection difficult and they were an infection risk to other duck or chicken flocks [9]. So, long-term control of the H5N1 panzootic in these areas may well depend on controlling the infection in duck flocks along with other strategies.

Recent field and experimental vaccines for HPAI in poultry include inactivated, conventional whole virus vaccines [3, 10, 11]; inactivated vaccines developed using reverse genetics [12–14]; in vitro baculovirus-expressed H5 antigen as subunit vaccines [10, 15]; in vivo vector-expressed vaccines including recombinant fowl poxvirus [16, 17], Newcastle Disease virus [18] or infectious laryngotracheitis virus vectors [19]; and DNA vaccines [20]. These vaccines have all conferred clinical protection and eliminated or reduced viral shedding following HPAI virus challenge. Most of these studies were carried out in chickens while those that were tested in ducks have also shown protective efficacy [12–14]. 

Use of H5 vaccination in ducks complicates serological surveillance using H5 antibody testing, as both infected and vaccinated ducks produce antibody to H5 haemagglutinin. Strategies to differentiate infected from vaccinated animals (DIVA) have been considered and developed for incorporation into surveillance programs for avian influenza in poultry [21]. In this study, we have evaluated two H5 vaccines in ducks that could potentially be used in DIVA strategies. One vaccine is a H5 subunit vaccine based on recombinant baculovirus expressed H5 haemagglutinin antigen. Serological differentiation of infected from vaccinated birds could be done by testing for antibody to viral antigens such as N1, M, NP, or NS1. The other vaccine is an inactivated H5N2 whole virus vaccine combined with tetanus toxoid antigen, with the latter antigen used as a positive marker of vaccination with this vaccine. These vaccines are compared with commercially available inactivated H5N2 whole virus vaccine in the ability to produce H5-specific antibody, to protect vaccinated ducks from a Vietnamese H5N1 HPAI virus and reduce virus shedding postchallenge.

## 2. Materials and Methods

### 2.1. Vaccines Used

 The recombinant baculovirus-expressed H5 vaccine was developed and produced at the Temasek Life Sciences Laboratory, Singapore (TLL vaccine) as described [22]. Briefly, three HA genes from different H5N1 clade 2.1 virus strains were amplified by RT-PCR and the resultant cDNA was inserted, through promoter-controlled expression cassettes, into baculovirus vectors before infecting insect cells. The vaccine product was obtained from the supernatant of infected insect cells and purified by ultracentrifugation. The purified supernatant was titrated for haemagglutinin (HA) activity using the method described previously [23], and the stock solution was diluted such that the antigen dose volume (0.2 mL) contained 2^8^ HA units per dose for the high-dose vaccine and 2^4^ HA units per dose for the low-dose vaccine. The adjuvanted vaccine preparations were then prepared by mixing the relevant H5 antigen concentration with an equal volume of commercial, water-in-oil adjuvant (Montanide, SEPPIC, Paris, France) to give a final dose of 0.4 mL per duck.

The inactivated H5N2 whole virus vaccine with tetanus toxoid marker antigen (TT/H5N2 vaccine) was prepared as a small pilot batch by Intervet International (Boxmeer, The Netherlands) using the standard H5N2 inactivated whole virus used in their Nobilis Influenza H5 vaccine combined with a commercial tetanus toxoid (TT) vaccine antigen (Pfizer, Melbourne, Australia) diluted to contain a final concentration of TT of 0.3 mg total protein per dose in the vaccine. This concentration had been shown to be highly immunogenic in previous studies when combined with inactivated H6N2 avian influenza virus in chickens and ducks [24, 25]. 

The above vaccines were compared with a commercially available inactivated H5N2 whole virus vaccine (Nobilis Influenza H5 vaccine, Intervet International Boxmeer, The Netherlands) (H5N2 vaccine). Both TT/H5N2 and H5N2 vaccines were formulated in a water-in-oil adjuvant. The dose of these vaccines given to the ducks was 1.0 mL which is twice the dose given to chickens on the manufacturers recommendation.

### 2.2. Source and Management of Ducks

The ducks used for these studies were young hybrid Pekin ducks (*Anus platyrhynchos*) from a commercial duck farm in Australia (Luv-a-Duck Farm, Nhill, Victoria, Australia). They were imported by air as day-old ducklings into Hong Kong after meeting quarantine approvals and health certification requirements in Australia. The farm of origin is free from avian influenza, Newcastle disease, duck plague (duck virus enteritis), and duck virus hepatitis viruses. Pre-export serological testing of the mother ducks for H5 and H7 avian influenza viruses by haemagglutination inhibition (HI) tests gave negative results. The ducklings were initially housed in the Department of Microbiology Animal House, University of Hong Kong (HKU) in pens with corn straw and wood shavings as bedding and given access to a water tub with continuous running water for bathing and drinking. Heat lamps were provided for rearing the young ducklings and they were given *ad lib* access to a commercial poultry grower pellet ration. Vaccinations and blood sampling were conducted in this animal house facility.

The ducks were transferred to the high biocontainment BSL3+ animal house facility of the State Key Laboratory of Emerging Infectious Diseases at HKU for the H5N1 virus challenge. The ducks were housed as vaccine groups in cages with absorbent bedding inside Class 3 negative-pressure, flexible film isolators with HEPA-filtered inlet air and exhaust air. Food and water were replenished daily and room lighting was on a 12-hour cycle. Animals were individually leg banded for identification. All animal experimentation was carried out with the approval of the institutional animal ethics committee and in compliance with the facility biosafety requirements. Researchers wore positive air pressure respirators and protective suits in the BSL3+ animal facility at all times.

### 2.3. Experimental Design

 An initial experiment was conducted with the TLL vaccine using groups of young ducklings vaccinated with either high dose TLL antigen (HD group, 5 ducks), high dose with adjuvant (HDA group, 5 ducks), or low dose with adjuvant (LDA group, 6 ducks) compared with mock vaccinated control group (6 ducks). For logistical reasons, there were insufficient 1 week old ducklings available for experiment 1, so two age groups of ducklings (1 week and 3 weeks old) had to be used for this preliminary experiment with TLL vaccine. These ducklings were randomly allocated to the vaccine groups or mock vaccinated control group. They were immunised subcutaneously at the nape of neck with the vaccine (0.2 mL of HA antigen stock with equal volume of adjuvant or diluent) and a second dose of the respective vaccine was given 3 weeks later. Four weeks after the second vaccination H5N1 virus challenge was conducted in BSL3+ animal house as described below. 

In the preliminary experiment with the TLL vaccine the challenge dose was based on a low dose challenge for H5N1 virus used in previous challenge studies in chickens but after completion of experiment 1 it was decided to use the higher H5N1 challenge dose that was more consistently used in other challenge studies with H5N1 in chickens and ducks using inactivated whole virus vaccines [10, 12]. 

In the second experiment, groups of young ducklings were vaccinated with high dose TLL vaccine with adjuvant (TLL HDA group, 8 ducks), TT/H5N2 vaccine (7 ducks) or H5N2 vaccine (7 ducks) and compared with an unvaccinated control group (7 ducks). The ducklings were immunised subcutaneously at the nape of neck at 1 week of age, with TLL vaccine given as a 0.4 mL dose and the TT/H5N2 and H5N2 vaccines given as a 1.0 mL dose. A repeat dose of the respective vaccine was given 3 weeks later. 

As postvaccination HI antibody response to the TLL vaccine was much weaker than for the whole virus vaccines and the challenge was to be at a higher virus dose, an additional higher dose of TLL vaccine (0.5 mL of HA antigen with equal volume of adjuvant) was given one week later (5 weeks of age) to this group. The H5N1 virus challenge was conducted in the BSL3+ animal house as described below when the birds were 9 weeks-old. 

In both experiments, the ducks were individually identified by leg bands and blood samples were collected prevaccination, after vaccination 1, vaccination 2 (prechallenge for TT/H5N2 and H5N2 groups), vaccination 3 (prechallenge for TLL group) and postchallenge. Serum samples in both experiments were tested for H5 antibody by HI tests and in the second experiment for antibody levels to TT by ELISA assays as described below.

### 2.4. Virus Challenge Procedure

 The challenge virus for both experiments was HPAI virus A/Vietnam/1203/04 (H5N1) (VN/1203/04), a clade 1 virus (WHO/OIE/FAO 2007), which was isolated from a human case early in the H5N1 epizootic, had been shown to be closely related genetically to H5N1 HPAI viruses from ducks and was shown to be highly pathogenic for ducks. Virus stocks were available at HKU. Preliminary titration (10-fold dilutions) of the virus stock was conducted in 7 week-old ducklings in the BSL3+ animal house to determine the challenge dose. The virus was administered by eye drop (100 *μ*L), intranasally (100 *μ*L) and orally (300 *μ*L) to each bird at a titre of 10^7.6^ EID_50_/mL (6 birds; dose = 10^7.3^ EID_50_), 10^6.6^ EID_50_/mL (5 birds; dose = 10^6.3^ EID_50_), 10^5.6^ EID_50_/mL (6 birds; dose = 10^5.3^  EID_50_) or 10^4.6^  EID_50_/mL (4 birds; dose = 10^4.3^  EID_50_). The 10^4.3^  EID_50_ dose (subsequently determined to be 21.6 duck LD_50_) caused mortality in all ducks within 7 days and this was selected as the challenge dose for the initial TLL vaccination experiment. A 10-fold higher dose (10^5.3^ EID_50 _ per bird), given via the same routes, was used for the second experiment as discussed under experimental design. 

In the BSL3+, animal house birds were anaesthetized by inhalation of isoflurane before being inoculated with 500 *μ*l VN/1203/04 virus at doses described above and were housed in separate BSL3 flexible film isolators. In the initial experiment, the unvaccinated controls and the LDA group were housed in cages in two separate smaller isolators and the HDA and HD groups were housed in cages in a larger isolator complex linked by a connecting chamber. In the second experiment, the unvaccinated controls and the TLL HDA group were housed in cages in the two separate smaller isolators and the TT/H5N2 and H5N2 groups were housed in cages in the larger isolator complex. In the initial TLL experiment, clinical signs and mortality were observed daily for 11 days and swabs were collected daily from the oropharynx and cloaca for virus culture. In the second challenge study, clinical signs and mortality were observed daily for 10 days and oropharyngeal and cloacal swabs were collected on alternate days for virus culture. Blood was collected from surviving birds at the end of both challenge studies to measure H5 antibody titres and for the second experiment to measure TT antibody levels.

Ducks dying after challenge in the second experiment were subjected to postmortem examination to observe the gross and histopathology changes after challenge with a Vietnamese H5N1 HPAI virus. Pathological examination, including immunoperoxidase staining of tissues from affected ducks, was conducted as described [3, 5]. 

### 2.5. Virus Isolation Procedures

Swab samples were collected into 1.0 mL aliquots of tissue culture medium 199 containing antibiotics (penicillin G (2 × 10^6^ U/L), polymyxin B (2 × 10^6^ U/L), gentamicin (250 mg/L), nystatin (0.5 × 10^6^ U/L), ofloxacin HCl (60 mg/L), and sulfamethoxazole (0.2 g/L)) and stored at −80°C. For virus isolation, samples were thawed, vortexed, centrifuged and the swab eluate was titrated in Madin Darby Canine Kidney (MDCK) cell cultures. Preliminary titration of the Vietnam/1203/04 virus showed that it replicated to the same titre in MDCK cells as it did in SPF chicken embryos. Briefly, swab eluate was serially diluted using half-log dilution in Gibco's modified essential medium (MEM) with 1% foetal calf serum using a 96-well microtitre plate. Each virus dilution was then transferred in quadruplicate to a 96-well MDCK confluent cell plate, with added 1% foetal calf serum in MEM. Cell cultures were incubated at 37°C for 3-4 days and examined daily for a cytopathic effect. The endpoint titres expressed as 50% tissue culture infective doses (TCID_50_) were determined [26].

### 2.6. Serological Procedures

Measurement of H5 antibody levels was conducted using the HI test procedure as described in the WHO Manual on Animal Influenza Diagnosis and Surveillance [27]. Briefly, 0.1 mL of test sera were incubated overnight at 37°C with an equal volume of commercial receptor destroying enzyme (RDE) and heat inactivated (56°C for 30 minutes) before further dilution to give a final 1 : 10 dilution. The sera were added to a 96-well microtitre plate and serially two-fold diluted, then incubated with 4 haemagglutinating units of inactivated H5 antigen (VN/1203/04) for 30–45 minutes at room temperature before the addition of 0.5% turkey red blood cells for 30 minutes incubation at room temperature. Turkey red cells are used in this laboratory as they were found to be more consistent and sensitive than chicken red cells. The antibody titre was expressed as the highest dilution giving complete inhibition of haemagglutination. Positive titres were interpreted as inhibition of haemagglutination at a serum dilution of 1 : 10 or greater.

Antibody to TT was measured by both a competitive ELISA (C-ELISA) and an indirect ELISA as described previously [25]. The antigen used for ELISA plate coating in both tests was purified tetanus toxoid (List Biological Laboratories, Inc., CA, USA) at optimized dilution in 0.05 M carbonate-bicarbonate buffer, pH 9.6. Briefly, in the C-ELISA, after thorough wash steps (6 times) in phosphate buffered saline pH 7.6/0.05% v/v Tween 20 (PBST), sequentially, duck sera diluted 1 : 10 in PBST/4% skim milk powder, then goat antitetanus toxoid antibody (Accurate Chemical & Scientific Corp., NY, USA) diluted 1 : 3200 in PBST/4% skim milk powder, then HRP conjugated chicken antigoat IgG diluted 1 : 20000 in PBST/4% skim milk powder (Chemicon, CA, USA) was incubated in washed plates for 60 minutes at 37°C. In the indirect ELISA, sequentially, duck sera at 1 : 200 dilution in PBS/4% skim milk powder, then mouse antiduck IgY antibody goat (SeroTec, UK) diluted 1:250 in PBS/4% skim milk powder, then HRP conjugated goat antimouse IgG (Santa Cruz Biotechnology, USA) diluted 1 : 20000 in PBS/4% skim milk powder was incubated in washed plates for 60 minutes at 37°C. After final washing in both tests TMB substrate (TMB One Solution, Promega Corp., WI, USA) was added for 5 minutes and stopped with 2 M sulphuric acid and plates were read in read at 450 nm with 630 nm reference wavelength using a spectrophotometer (Dynex MRX II, Chantilly, VA, USA). Each plate included 8 replicates of negative control and duplicates of positive control sera from 4 ducks that had been vaccinated with 2 doses of commercial tetanus toxoid vaccine. In the C-ELISA the level of TT-specific antibodies was calculated using the formula: % Inhibition = 100 − [100× (Mean OD test serum/Mean OD negative control serum)]. The cut-off, as determined previously [25] was 30% inhibition. 

In the indirect ELISA the antibody level was reported as a percentage of the positive = 100 × (mean OD test-mean OD negative)/(mean OD positive-mean OD negative). The cut-off was based on mean + 3 × standard deviations of 50 negative ducks (1 to 9 weeks-old) and calculated to be 18.5% of mean OD positive control.

### 2.7. Statistical Analysis

The geometric mean titre (GMT) of the level of virus shed via cloaca and oropharynx from individual birds in each group was determined for consecutive days postchallenge and compared between groups by ANOVA. In groups where deaths occurred the GMT was determined for the remaining group members. The GMT of H5 HI antibody responses and the ELISA antibody responses to TT antigen were compared within and between groups pre- and postvaccination and pre- and postchallenge by ANOVA

## 3. Results

### 3.1. Protection from Disease by the Vaccines

In the preliminary study with the TLL vaccine at lower challenge dose, mild clinical signs of ocular discharge were noticed on day 2 and by day 3; mild ocular discharge or conjunctivitis was evident in 5/6 control and 5/5 HD ducks but only 1/6 LDA and 1/5 HDA ducks. Also by day 3 control ducks started to become depressed, unkempt with reduced grooming, and reduced interest in feed; one developed neurological signs (head tilt, head shake, tremors) and was found dead on day 4; another had neurological signs on day 5 and was euthanized; two others on day 7 and another on day 8 were moribund and were euthanized. The remaining control duck was observed with a mild head tilt on day 8 but continued to feed until the end of experiment on day 11. One HD duck was found dead on day 7. All HDA (5) and LDA (6) ducks and the other HD (4) ducks were healthy, active, alert and consuming feed until the completion of the experiment (Table 1).

In the second phase of the study, with TLL HDA, H5N2/TT and H5N2 vaccinated birds and controls given the higher challenge dose, signs of ocular discharge were noticed on day 1 in a couple of control ducks and by day 2, 4/7 control birds had mild to copious eye discharge while only mild discharge was present in 1/8 TLL HDA vaccinated duck and in 1/7 H5N2 vaccinated birds. Two controls were found dead on day 2 and 3, respectively; one other showed neurological signs and another had a swollen, oedematous head on day 4 and both were found dead on day 5; two others developed mild ataxia and incoordination and another developed conjunctivitis on day 5, one of these became more severely ataxic although continuing to feed and was euthanized on day 9 and the remaining 2 control ducks had continued to feed but had neurological signs including incoordination and head tilt on day 10 when they were euthanized and the experiment was completed. In contrast, none of the TLL HDA vaccinated birds died or showed neurological disease signs, one H5N2 vaccinated duck was found dead on day 7 and one H5N2/TT vaccinated duck was found dead on day 8 (Table 2). The other vaccinated ducks remained healthy, were active and alert and continued to eat and drink.

### 3.2. Virus Isolation Results

In the preliminary trial with the TLL vaccine, oropharyngeal virus shedding occurred in control ducks from the first day and continued for 5 days. On days 3 and 4, virus was detected from the oropharynx of all 6 ducks at titres between 10^3.0 ^and 10^5.0^ TCID_50_/mL. For the HDA and LDA vaccine groups' virus was detected from the oropharynx of 1/5 or 3/6 ducks, respectively, on day 1 but not any subsequent day. In contrast for the HD group, virus was shed intermittently from the oropharynx from 2/5 ducks from day 1 to day 4 at titres ranging from 10^1.79 ^to 10^3.21^ TCID_50_/mL; and persistently from day 2 to day 7 from one duck (that subsequently died on day 7) at titres ranging from 10^2.12^ to 10^3.37^ TCID_50_/mL. Nonetheless, the mean virus titres in oropharyngeal swabs for the HD group remained significantly lower (*P* < .05) than for the control ducks on days 2 to 5 postchallenge. In comparison to the virus recovery from the oropharynx, no virus could be isolated from the cloacal swabs of any challenged ducks in this experiment. 

In the comparative study with TLL HDA, H5N2 and H5N2/TT vaccines at the higher challenge dose, virus was detected in the oropharynx of control ducks between day 2 and day 5 at titres ranging from 10^3.3^ to 10^4.6^ TCID_50_/mL. In comparison to the virus recovery from the oropharynx, virus could only be sporadically isolated from the cloacal swabs at low titre (10^1.9^ TCID_50_/mL) from two control ducks, one on day 2 and one on day 5 postchallenge. By contrast, no virus could be detected from the oropharyngeal swabs or cloacal swabs from any duck in the TLL HDA, H5N2 or H5N2/TT groups throughout this experiment. The virus isolation results on day 2 and day 4 postchallenge for both experiments are summarised in Tables 1 and 2.

### 3.3. H5 Antibody Responses

In the preliminary TLL vaccine study, only 2/5 ducks from the HDA vaccine group showed weak H5 HI antibody responses (1 : 10 and 1 : 20) against VN/1203/04 antigen prior to challenge. None of the LDA, HD or control ducks had detectable antibody against VN/1203/04 prior to challenge. By day 11 postchallenge, most vaccinated ducks (12/15) showed a greater than four-fold increase in HI antibody titre against VN/1203/04. There were no significant difference in H5 HI GMT between HDA, LDA and HD groups postchallenge. The surviving control duck showed a postchallenge titre of 1 : 320. 

In the comparative study with TLL HDA, H5N2 and H5N2/TT vaccinations, only 3/8 TLL HDA vaccinated ducks after the second vaccination showed low H5 HI antibody titres (1 : 10, 1 : 20 and 1 : 40) against VN/1203/04. After the third TLL vaccination (i.e., prechallenge) 6/8 ducks showed low (5 ducks at 1 : 10) or moderate (1 duck at 1 : 160) antibody titres to VN/1203/04. In contrast, all 7 H5N2 and all 7 H5N2/TT ducks had antibody to VN/1203/04 (HI titres from 1 : 20 to 1 :  640) after two vaccinations (i.e., prechallenge). The H5 HI GMT for the TLL HDA group prechallenge were significantly lower than H5N2 group (*P* < .01) and the H5N2/TT group (*P* < .05). H5 HI antibodies against VN/1203/04 were not detectable in control ducks prior to challenge. After VN/1203/04 challenge, the TLL HDA vaccine and the H5N2/TT vaccine groups showed a greater than four-fold rise in HI GMT (TLL 1 : 16→1 : 160; H5N2/TT 1 : 49→1 : 422) while H5N2 vaccine group showed only a slight rise in HI GMT (1 : 119 → 1 : 160). There were no significant differences between the postchallenge H5 HI GMT between TLL HDA, H5N2 and H5N2/TT vaccine groups. The two surviving control ducks showed either an undetectable titre (<1 : 10) or a titre of 1 : 2560. The H5 HI serology results for both experiments are summarised in Tables 1 and 2.

### 3.4. TT Antibody Responses

None of the ducks in the TLL HDA, H5N2 and H5N2/TT vaccine groups or the control ducks had antibody to TT antigen in prevaccination serum samples, or in subsequent prechallenge or postchallenge samples from the TLL HDA or H5N2 vaccine groups, or controls with either the TT C-ELISA or the TT indirect ELISA. 

In the H5N2/TT vaccine group after the first vaccination 3/7 ducks were positive in the TT C-ELISA and 1/7 ducks was positive by TT indirect ELISA; prechallenge all ducks were positive by TT C-ELISA and TT indirect ELISA; and postchallenge all were positive by TT indirect ELISA but 2 ducks were negative by TT C-ELISA. The mean % inhibition or % of mean positive controls OD, standard deviations for the C-ELISA and the indirect ELISA, respectively, and significance of differences at different time points are shown in Table 3.

### 3.5. Postmortem Findings

Postmortem examination of dead control ducks from the second experiment revealed gross lesions of congestion and haemorrhage in multiple organs. These affected organs included the lungs, heart, pancreas, intestines, liver, spleen, kidney, and bursa. Increased pericardial fluid with fibrin clots, patches of pallor in the heart ventricle, areas of mottling and necrosis in pancreas, moist appearance of viscera, carcass emaciation and dehydration, and congested blood vessels in the brain were evident in some cases. 

From the histopathology examination of tissue sections, the control ducks that died showed varying combinations of the following changes: congestion and oedema in the lungs; lymphohistiocytic tracheitis and necrosis of tracheal epithelial cells in some cases; congestion and multiple small foci of glial cell and neurone necrosis and/or multifocal lymphohistiocytic meningo-encephalitis, with some perivascular cuffing and gliosis in the brain; lymphohistiocytic myocarditis and multifocal myocardial necrosis in some cases; multifocal pancreatic necrosis; congestion and small foci of hepatic periportal lymphocyte necrosis; congestion and lymphoid depletion in the spleen and general viscera congestion. 

Immunoperoxidase staining for H5 HA antigen in lung, brain, spleen, kidney, pancreas and heart sections for all control ducks and bursa, thymus and trachea in some control ducks confirmed the presence of H5N1 infection in these tissues.

However, gross and histopathological examination of the H5N2 duck that died on day 7 and the H5N2/TT duck that died on day 8 did not show lesions that were consistent with the above pathology that has been recorded previously for H5N1 HPAI in ducks [5]. Both ducks had multifocal ulcerative superficial pyogranulomatous proventriculitis with presence of gram-negative cocco-bacilli, heterophil infiltration in the spleen and multifocal mild to moderate tubular nephrosis suggesting death from non-H5N1 causes. Immunoperoxidase staining of lung, brain, spleen, kidney, pancreas, heart, bursa, liver, trachea, and proventriculus for H5 HA antigen in sections from the dead H5N2 and H5N2/TT ducks gave uniformly negative results.

## 4. Discussion

The preliminary study showed that the TLL baculovirus-expressed H5 vaccine was able to protect ducks from severe disease and mortality following challenge from a dose of 10^4.3^EID_50_ of H5N1 virus (Vietnam/1203/04), that is highly pathogenic for ducks. All ducks immunised with low-dose or high-dose vaccines administered with water-in-oil adjuvant were protected but efficacy was lower in the ducks immunised with high-antigen dose without adjuvant, underlining the role of adjuvants in inactivated avian influenza vaccines [1]. Virus reisolation results further indicated that the birds vaccinated with the baculovirus H5 vaccines only excreted virus briefly and oropharyngeal virus shedding was eliminated (for HDA or LDA groups) compared to unvaccinated control birds. 

The comparative vaccination study showed that the TLL HDA vaccine, given as a three dose regime and the TT/H5N2 marker vaccine (2 doses) were as effectively as commercial H5N2 avian influenza vaccine (2 doses) in protecting ducks from severe disease and mortality following challenge with a ten-fold higher dose (10^5.3^EID_50_) of H5N1 virus (Vietnam/1203/04). All three vaccines showed a similar high level of efficacy in preventing virus shedding from oropharynx and cloaca compared with control ducks. This is a considerable advantage for the control of virus transmission from duck to duck or to other poultry species in the field and in reducing the environmental virus load. 

The prechallenge serological responses to H5 HA for the ducks vaccinated with inactivated whole H5N2 virus and TT/H5N2 vaccines are similar to those reported previously with inactivated whole virus H5 vaccines [12] but the prechallenge H5 antibody response to TLL vaccine was poor and low levels of HI antibody was only detected in some ducks given two or three doses of the TLL HDA vaccine. However, despite low serum H5 antibody responses the TLL vaccinated birds remained protected against HPAI challenge. In both the preliminary study and in the comparative study, TLL vaccinated ducks mounted ≫4-fold rise in H5 antibody titre after challenge. The TT/H5N2 group also showed a >4-fold rise in H5 titre after challenge but the H5N2 group did not, however there were no significant differences in postchallenge titres between TLL, H5N2 and TT/H5N2 vaccine groups. Other studies have reported that despite the absence of detectable HI antibodies (<1 : 10) in the prechallenge sera of ducks vaccinated with either of the two lowest vaccine doses of a reverse genetics H5N3 vaccine, there was no virus shedding or disease or deaths after challenge with a duck-lethal H5N1 virus [12].

In nature, replication of avian influenza viruses can occur in infected ducks without significant serum antibody response, but despite a poor antibody response, ducks were immune and could resist virus reinfection [28, 29]. Protection in the vaccinated ducks against HPAI, despite the absence of HI antibody titres, may be due to a priming of duck secretory or mucosal immunoglobulins [30], or cell-mediated immunity [31]. The lower prechallenge serum antibody response in TLL ducks may be a function of too low an antigen load in the vaccine for use in ducks compared with the dose required for chickens, which has been discussed previously [31] and recognised by the manufacturer of the inactivated H5N2 vaccine, who advised use of 1.0 mL dose in ducks instead of the standard 0.5 mL dose in chickens. Another consideration with the baculovirus expressed H5 antigen is that possible differences in glycosylation of insect versus avian cells may have contributed to the low H5 HI antibody titres in vaccinated ducks. 

The more frequent reisolation of H5N1 virus in challenged ducks from the oropharynx and only sporadic isolation from cloacal swabs after challenge with A/VN/1203/04 H5N1 HPAI is consistent with previous studies where viral titres shed from the trachea of ducks were higher than from the cloaca for Eurasian H5N1 viruses since 2003 and is believed to be related to a shift in replication efficiency for the upper respiratory tract [4, 6, 32]. 

These challenge studies were conducted using a heterologous H5N1 virus strain and it could be expected that these vaccines would remain efficacious when used in geographical areas where different H5N1 virus clades exist. In contrast to human influenza vaccines, vaccines for poultry do not appear to require close antigenic homology with the haemagglutinin protein and remain able to offer broad cross-protection against diverse field viruses. For example, a single recombinant fowlpox-H5 vaccine was able to clinically protect chickens from challenge by nine different HP H5 strains that had between 87.3 and 100% HA protein sequence similarity with the vaccine strain [2]. In this study, ducks were challenged with a heterologous clade 1 H5N1 virus and resisted disease and death despite only 94-95% HA1 protein sequence similarity between vaccine and virus strains. However, some recent clade 2.3.2 and clade 2.3.4 H5N1 viruses have shown substantial antigenic diversity from the contemporary H5N1 viruses [33] and the efficacy of the TLL H5 subunit and H5N2 vaccines used in this study would need to be tested specifically against these new viruses before use in control programmes for them.

Ducks vaccinated with the TT/H5N2 vaccine produced TT antibody responses similar to those in ducks vaccinated with TT/H6N2 vaccine in previous studies [25]. Antibody responses after the initial vaccination with the TT/H5N2 vaccine were detected in more ducks by C-ELISA (3/7) than indirect ELISA (1/7) but after second vaccination (prechallenge) all ducks were TT antibody positive (prechallenge) at similar levels of reactivity by C-ELISA and indirect ELISA. After H5N1 challenge TT antibody responses by indirect ELISA persisted at similar levels to prechallenge in all ducks, but antibody levels measured by C-ELISA were reduced and two ducks were below the test cut-off. This observation was similar to findings with a previous study with an in-house TT/H6N2 vaccine in Muscovy ducks where indirect ELISA TT antibody persisted at high levels from 6 to 19 weeks postvaccination but C-ELISA results on the same serum showed some reduction in TT antibody response [25]. Therefore, the indirect ELISA would be better for field use to monitor TT antibody if a H5/TT marker vaccination was being used. 

While vaccination remains an important disease control tool for avian influenza, experiences in Hong Kong, Italy, USA and elsewhere showed that vaccination should be part of a programme that incorporates use of quality and efficacious vaccines; quarantine, movement restriction, depopulation and disposal of affected flocks; application or enhancement of flock biosecurity in farms and markets; surveillance to monitor vaccine efficacy as well as field virus circulation; and public awareness on disease prevention and control [3, 11, 34]. Success with vaccination programs for control of H5N1 HPAI has been quite variable in poultry in East and Southeast Asia, owing to difficulties such as: administering a correct dose to individual birds that are freely roaming and hard to catch; providing an adequate coverage at regular intervals and being inclusive of new hatchlings or purchased birds; maintaining cold chain in tropical climates; observing biosecurity in a village setting; and challenges that local manufacturers may face in ensuring antigen yield, inactivation of killed vaccine and formulation with adjuvants. Specific issues relating to vaccine use for control of H5N1 HPAI includes the risk that vaccination may allow ongoing H5N1 transmission from vaccinated but inapparently infected birds and whether vaccine use may promote antigenic drift and lead to H5N1 virus endemnicity [35]. These concerns especially relate to H5N1 infection in ducks which are more likely to survive virus infection and produce H5 antibody levels of the same order as ducks that are vaccinated with inactivated whole virus H5 vaccines.

The two vaccines evaluated in this study have shown equivalent efficacy to a commercial inactivated whole virus H5N2 vaccines in ducks, but also have some additional features that may enhance their effectiveness for field use in village poultry avian influenza control systems in developing countries. The recombinant H5 baculovirus vaccine has the major advantage that it does not require embryonated chicken eggs or a high biocontainment facility for production. It should also be relatively straightforward to alter the H5 insert in the baculovirus to match an evolving field H5N1 virus strain. This is a subunit vaccine and after 3 doses in this study produced only weak H5 antibody responses but protected ducks from disease and very significantly reduced virus shedding in H5N1 challenged ducks. The only qualifier with this vaccine is that the third vaccine dose at higher antigen load did produce increased H5 antibody response and this vaccine should be used at a higher antigen dose in ducks, which may induce stronger H5 HI antibody responses. Vaccinated and infected ducks developed an anamnestic antibody response to H5 HA after challenge but because it is a HA subunit vaccine, testing for antibody to influenza A proteins like NP, NS1 or M does confirm the presence of active infection and can be used as a DIVA strategy [21]. 

The TT/H5N2 marker vaccine is as efficacious as the commercial H5N2 vaccine and has the advantage of positively identifying vaccinated ducks. Serological surveillance of ducks for vaccination effectiveness and evidence of virus incursion is particularly difficult where accurate farm records or physical identification of vaccinated birds (leg or wing bands) are not available. Ducks vaccinated with this TT/H5N2 marker vaccine could be very effectively monitored for epidemiological purposes by simple ELISA and HI tests for TT antibody and H5 antibody, respectively. For example, if this vaccine was the approved vaccine for the district or region, simply testing a statistically appropriate sample by TT ELISA would determine the effectiveness of the coverage by the approved vaccine; concurrent testing of H5 antibody would monitor the potency of the vaccine in the field and whether the vaccine handling, storage and application was producing expected levels of flock immunity, and facilitate investigation of poor vaccine responses; testing for TT and H5 antibody during investigation of H5N1 outbreaks in vaccinated flocks would confirm that affected birds had, or had not been effectively vaccinated, and if the vaccine was not effective against the new circulating antigenic strains of the virus; and provide objective measures of risk for H5N1 cases in the district or region based on the level of flock immunity to allow prioritization of avian influenza control activities. Additionally, TT/H5N2 vaccinated ducks in this study, that were challenged with H5N1 viruses, showed significant rises in H5 antibody titre that could provide a signal of recent virus infection. Similar rises in H5 antibody titre have been observed in TT/H5N2 vaccinated chickens after challenge with H5N1 HPAI (Dr Deborah Middleton, CSIRO-AAHL, Geelong, personal communication). Field use of this vaccine could establish normal antibody response curves for TT and H5 antibodies in vaccinated, uninfected ducks. Higher than expected H5 antibody titres, indicating an anamnestic response, would then require further virological investigation. 

Extensive vaccination programs for control of H5N1 HPAI have been or are currently being conducted in countries like Vietnam, Indonesia, China and Egypt but outbreaks are still occurring in village poultry systems with domestic ducks being implicated in virus persistence. The two novel vaccines evaluated in this study show equivalent efficacy to an existing commercial vaccine in ducks but they offer advantages for surveillance in village poultry systems in the above counties. The baculovirus recombinant H5 vaccine could be further developed and optimally manufactured in standard vaccine facilities in a developing country without the need for large scale chicken embryo culture facilities. The TT marker can be readily incorporated into any inactivated whole virus or subunit H5 vaccine and used in H5 vaccination programs to provide an effective positive marker of vaccination for surveillance of the small scale and village poultry industries. These tools are recommended for consideration as part of vaccination control programs in countries with ongoing problems of H5N1 HPAI control.

## Figures and Tables

**Table 1:** Efficacy of TLL H5 vaccine in ducks challenged with avian influenza virus [A/VN/1203/04 (H5N1)].

Vaccine group^1^	Challenge dose (EID_50_)	Morbidity^2^	Mortality^2^ (Mean time to death)	HI serology^3^		Virus isolation-oropharyngeal^4^		Virus isolation-cloacal^4^	
				Prechallenge No.>HI 10 (GMT > HI 10)	Postchallenge No.>HI 10 (GMT > HI 10)	Day 2	Day 4	Day 2	Day 4
Control	10^4.3^	1/6^2^	5/6 (6 days)	0	1 (320)	4/6 (2.27)	6/6 (3.66)	0/6	0/5
TLL H5 High-dose	10^4.3^	0/5	1/5 (day 7)	0	4 (452)	1/5 (0.42)	3/5 (1.87)	0/5	0/5
TLL H5 Low-dose adjuvant	10^4.3^	0/6	0/6	0	5 (184)	0/6	0/6	0/6	0/6
TLL H5 High-dose adjuvant	10^4.3^	0/5	0/5	2 (14)	3 (508)	0/5	0/5	0/5	0/5

^1^The TLL vaccine was given as 2 doses at 3 weeks interval with challenge 4 weeks later. Challenge was with 10^4.3^ EID_50_ of the H5N1 virus.

^2^Number affected/total challenged. Morbidity refers to neurological signs of head tilt, ataxia and weakness.

^3^Haemagglutination inhibition (HI) test results using A/VN/1203/04 (H5N1) antigen showing the number of ducks positive with HI titre > 10 and the geometric mean titre (GMT) for the positive ducks is shown in parenthesis.

^4^Number shedding virus/number alive at each time point. Values in parenthesis are mean viral titres expressed in log⁡_10_ TCID_50_/mL.

**Table 2:** Efficacy of H5 vaccines in ducks challenged with avian influenza virus [A/VN/1203/04 (H5N1)].

Vaccine group^1^	Challenge dose (EID_50_)	Morbidity^2^	Mortality^2^ (Mean time to death)	HI serology^3^		Virus isolation-oropharyngeal^4^		Virus isolation-cloacal^4^	
				Prechallenge No.>HI 10 (GMT > HI 10)	Postchallenge No.>HI 10 (GMT > HI 10)	Day 2	Day 4	Day 2	Day 4
Control	10^5.3^	2/7 ^2^	5/7 (4.8 days)	0	1 (2560)	7/7 (3.21)	5/5 (3.42)	1/7 (0.04)	0/5
TLL H5 High-dose adjuvant	10^5.3^	0/8	0/8	6 (16)	8 (160)	0/8	0/8	0/8	0/8
Inactivated H5N2 vaccine.	10^5.3^	0/7	1/7 (day 7)	7 (119)	6 (160)	0/7	0/7	0/7	0/7
Inactivated H5N2 with TT	10^5.3^	0/7	1/7 (day 8)	7 (49)	6 (422)	0/7	0/7	0/7	0/7

^1^ The TLL vaccine was given in 3 doses 1, 4 and 5 weeks of age, the other vaccines were given at 1 and 4 weeks of age and challenge was at 9 weeks of age with 10^5.3^ EID_50_ of the H5N1 virus.

^2^ Number affected/total challenged. Morbidity refers to neurological signs of head tilt, ataxia, and weakness.

^3^ Haemagglutination inhibition (HI) test results using A/VN/1203/04 (H5N1) antigen showing the number of ducks positive with HI titre > 10 and the geometric mean titre (GMT) for the positive ducks is shown in parenthesis.

^4^ Number shedding virus/number alive at each time point. Values in parenthesis are mean viral titres expressed in log⁡_10_ TCID_50_/mL.

**Table 3:** Antibody responses to tetanus toxoid antigens in ducks vaccinated with H5N2/TT marker vaccine and challenged with avian influenza virus [A/VN/1203/04 (H5N1)].

TT ELISA type	Post-vacc. 1	Post-vacc. 2	Postchallenge
	Mean (Std Dev.)^3^	Mean (Std Dev.)	Mean (Std Dev.)
TT C-ELISA	30.7%	62.4%	35.7%
(% inhibition)^1^	(9.5%)^a^	(9.2%)^b^	(17.3%)^a^
TT Indirect ELISA	7.7%	65.4%	67.7%
(% positive)^2^	(7.4%)^c^	(28.8%)^d^	(31.8%)^d^

^1^The positive-negative cut-off point for the TT C-ELISA is 30% inhibition [25].

^2^The positive-negative cut-off point for the TT indirect ELISA was 18.5% of mean OD of the positive control.

^3^Within the different TT ELISA test rows, the mean results at different times postvaccination or postchallenge with different letter superscripts (^a, b^ or ^c, d,^) are significantly different (*P* < .05).

## Abbreviations

AI: Avian influenza
ANOVA: Analysis of variance
C-ELISA: Competitive enzyme linked immunosorbent assay
DIVA: Differentiating infected from vaccinated animals
EID_50_,: 50% egg infectious dose
FAO: Food and Agricultural Organisation
GMT: Geometric mean titre
HA: Haemagglutinin
HD: High dose
HDA: High dose with adjuvant
HI: Haemagglutination inhibition
HPAI: Highly pathogenic avian influenza
LDA: Low dose with adjuvant
LPAI: Lowly pathogenic avian influenza
MDCK: Madin Darby canine kidney cells
NA: Neuraminidase
NP: Nuclear protein
NS1: Nonstructural protein
OIE: World Organisation for Animal Health
PBST: Phosphate-buffered saline/Tween 20
TLL: Temasek Life Sciences Laboratory
TT: Tetanus toxoid
WHO: World Health Organisation.
